# Discrimination of Deoxynivalenol Levels of Barley Kernels Using Hyperspectral Imaging in Tandem with Optimized Convolutional Neural Network

**DOI:** 10.3390/s23052668

**Published:** 2023-02-28

**Authors:** Ke-Jun Fan, Bo-Yuan Liu, Wen-Hao Su

**Affiliations:** College of Engineering, China Agricultural University, Beijing 100083, China

**Keywords:** hyperspectral imaging, deoxynivalenol, feature wavelength selection, convolutional neural network

## Abstract

Deoxynivalenol (DON) in raw and processed grain poses significant risks to human and animal health. In this study, the feasibility of classifying DON levels in different genetic lines of barley kernels was evaluated using hyperspectral imaging (HSI) (382–1030 nm) in tandem with an optimized convolutional neural network (CNN). Machine learning methods including logistic regression, support vector machine, stochastic gradient descent, K nearest neighbors, random forest, and CNN were respectively used to develop the classification models. Spectral preprocessing methods including wavelet transform and max-min normalization helped to enhance the performance of different models. A simplified CNN model showed better performance than other machine learning models. Competitive adaptive reweighted sampling (CARS) in combination with successive projections algorithm (SPA) was applied to select the best set of characteristic wavelengths. Based on seven wavelengths selected, the optimized CARS-SPA-CNN model distinguished barley grains with low levels of DON (<5 mg/kg) from those with higher levels (5 mg/kg < DON ≤ 14 mg/kg) with an accuracy of 89.41%. The lower levels of DON class I (0.19 mg/kg ≤ DON ≤ 1.25 mg/kg) and class II (1.25 mg/kg < DON ≤ 5 mg/kg) were successfully distinguished based on the optimized CNN model, yielding a precision of 89.81%. The results suggest that HSI in tandem with CNN has great potential for discrimination of DON levels of barley kernels.

## 1. Introduction

Barley (*Hordeum vulgare* L.), which is the fourth most grown cereal in the world [1], is an ancient and significant cereal grain crop [2]. While barley is suffering from many diseases due to global climate change. Among these diseases, Fusarium head blight (FHB), caused mainly by Fusarium, has rapidly become one of the devastating crop diseases worldwide [3]. The three main negative effects of FHB infection in cereals include impaired technical quality, loss of cereal yield and contamination with Fusarium toxins [4]. During infection, Fusarium spp. can produce a number of trichothecene toxins, such as deoxynivalenol (DON), nivalenol (NIV) [5], zearalenone (ZEA) and moniliformin (MON) [6]. Of these trichothecene mycotoxins, DON is the most common one in barley, which can cause food refusal, vomiting, diarrhea and dermatitis [7]. DON content is routinely measured before the harvested grain for malt, food, and feed [8]. Regulatory guidelines for DON levels vary by country [9]. The US Food and Drug Administration has set a recommended limit of 1.00 mg/kg for DON in processed wheat products [10], while Brazil has established DON limits of 1.00 mg/kg and 0.75 mg/kg in whole wheat grain and other wheat derivates, respectively [11]. In addition, the maximum level of DON in unprocessed grains has been approved by the European Union at 1.25 mg/kg [12]. Therefore, cereals with DON levels higher than 1.25 mg/kg should not be used as food for humans. However, it can be mixed with clean cereals or other cereal batches low in DON to eventually produce cereal batches for feed specifications.

Due to the devastating nature of FHB, it is vital for growers to devise strategies to mitigate losses caused from the disease [13]. These strategies include cultural practices, tolerant cultivars or planting resistant, biological control, chemical control, harvesting strategies, and use of forecasting systems. Agronomic practices and fungicides only partially reduce the risk of damage [14]. The best way to control FHB and reduce mycotoxin contamination is to create wheat genotypes carrying effective resistance genes [15]. Thousands of breeding lines are screened for DON levels annually to develop resistant cultivars. The resistance to DON accumulation in barley is inherited quantitatively [8]. Due to the health concerns, barley replaced wheat cultivated area, food or feed requires periodic detection of DON levels. And the barley kernels are harvested for DON assays. Nowadays, various techniques have been used to detect and quantify DON contamination in cereals. The typical chemical methods include gas chromatography-mass spectrometry (GC-MS), high performance liquid chromatography (HPLC), and enzyme-linked immunosorbent assay (ELISA). Although these techniques can determine the DON concentration, they are time consuming, costly, and destructive [16]. Consequently, farmers and the food industry need to seek alternative techniques for rapid and non-destructive detections of DON levels.

Spectroscopic methods have been used to evaluate the mycotoxins in cereals widely [17]. Hyperspectral imaging (HSI) is an emerging, non-destructive and cost-effective method for capturing spectral data at a per-pixel location in a sample image. This method may become an alternative to time-consuming wet chemical methods for toxin assessment [18]. However, one of the challenges in HSI is how to handle large amounts of high-dimensional data. Traditional machine learning algorithms have been widely used in the field of spectral data analysis. Hamidisepehr and Sama [19] tested several commercial machine learning algorithms using spectral data collected from moisture-controlled silt loam and wheat straw residue samples. The stereoscopic support vector machine (SVM) and integrated bagged tree methods predicted 96% and 93% accuracy for the silt samples and 86% and 93% accuracy for the wheat straw residue samples respectively. Feng, et al. [20] used three different data fusion approaches (raw data fusion, feature fusion and decision fusion) to fuse three types of spectral features for the classification of rice diseases using machine learning methods such as SVM and logistic regression (LR) with the accuracy of over 93%.

The performance of detection of models built by conventional algorithms may be limited by specially designed constraints and model parameters. Deep learning has been proven to be an advanced big data analysis technique applied in food quality inspection [21]. Yu, et al. [22] presented a novel method for the non-destructive identification of pesticide residues on the surface of cantaloupe using visible/near infrared (Vis/NIR) spectroscopy (348 -1141 nm) in combination with deep feature fusion. The proposed one-dimensional convolutional neural network (1D-CNN) model accurately distinguished the presence of pesticide residues with an identification accuracy of 99.17%. Later, Zhu, et al. [23] implemented a rapid in situ identification of pesticide residues in tea by combining surface-enhanced Raman scattering (SERS) and 1D-CNN. In recent years, 1D-CNN has made some progress in the non-destructive detection of agricultural products by combining with spectral imaging techniques. Gao, et al. [24] used 1D-CNN to classify the presence of aflatoxin in peanuts and wheat with the highest test accuracy of 96.35%. A model based on HSI and 1D-CNN to differentiate soybean seed varieties achieved a classification accuracy of over 98% [25]. Another method based on Vis/NIR spectroscopy combined with 1D-CNN was proposed for the non-destructive detection of pesticide residues on the surface of cantaloupe with an accuracy of 95.83% [22]. To the best of our knowledge, no previous study has been conducted to assay the DON content in barley kernel samples combining hyperspectral techniques and 1D-CNN. Furthermore, feature variable selection allows the best combination of several discrete wavelengths to be determined from hundreds of spectral variables, which is a straightforward and effective way to reduce redundant data [26,27,28,29]. Wavelength selection approaches like competitive adaptive reweighted sampling (CARS), successive projections algorithm (SPA), first order derivative and mean centre iterative algorithm (FMCIA) and uninformative variable elimination (UVE) have been effectively employed to recognize information on characteristics related to chemical composition, texture, authenticity and fungal contamination of food products [30,31].

In this study, 1D-CNN algorithm was employed to analyze the obtained hyperspectral data to assay the DON content in barley kernels. The specific objectives were to: (1) evaluate the potential of HSI for DON level detection in barley grain; (2) construct a deep learning model on the basis of the 1D-CNN algorithm and to compare its capabilities with conventional machine learning models; (3) build a classification algorithm to distinguish samples with the low DON level (≤1.25 mg/kg) from those with higher DON levels.

## 2. Materials and Methods

### 2.1. Data Collection

Barley samples used in this study varied depending on their reaction to FHB. Samples were taken from field plots that produced artificial epidemics of FHB. The grain spawning method proposed by Steffenson [32] was used for inoculation. The spikes of each strain were harvested at physiological maturity in August, desiccated to approximately 2% moisture in a forced air dryer at 12 °C for 35 days, threshed and cleaned. After the resulting cereal samples were used for hyperspectral scanning imaging, the DON content (mg/kg) of barley samples is assayed by triplicate analysis using the Gas Chromatograph-Mass Spectrometer (GC-MS) reference method [33]. A total of 590 samples were evaluated (about 10 g per sample). Based on the DON levels determined by GC-MS, the samples in the calibration group were divided into 3 categories: 28 samples in class I (0.19 mg/kg ≤ DON ≤ 1.25 mg/kg), 109 samples in class II (1.25 mg/kg < DON ≤ 5 mg/kg) and 453 samples in class III (5 mg/kg < DON ≤ 14 mg/kg).

**Figure 1:**
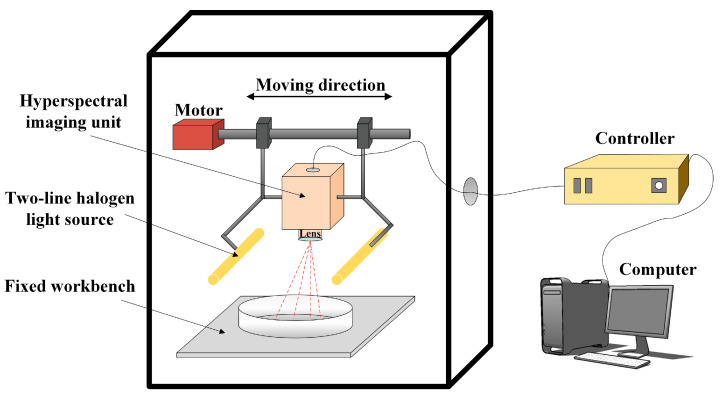
Diagram of the hyperspectral imaging (HSI) system.

Before GC-MS analysis, hyperspectral images of the barley samples were obtained by a linear scan Vis/NIR HSI system (367–1048 nm). As shown in Figure 1, this hyperspectral system (PlantSpec 10, Middleton Spectral Vision, Middleton, WI, USA) consists of a complementary metal oxide semiconductor (CMOS) (MSV500, Middleton Research, Middleton, WI, USA), a two-line halogen light source (2 × 13 × 35 W, luminous flux: 840–950) (MSV Series 13 illumination, Middleton Spectral Vision, Middleton Spectral Vision, Middleton, WI, USA), a spectrometer with optical resolution (V10E, Specim, Oulu, Finland) consisting of a 1.2 nm, a computer with data acquisition software for controlling the hyperspectral camera, scanner carrier table and image acquisition, and a conveyor system operated by stepper motors. Once all hyperspectral images were acquired, a developed coding algorithm was used to calculate the calibration of all spectral images. The average spectrum of each sample was then automated and extracted according to Matlab R2019a (The Mathworks Inc., Natick, MA, USA). Data from the hyperspectral images were restricted to the interval 383–1030 nm (550 variables) due to the low signal-to-noise ratio on both sides of the obtained spectra. The number of the samples in this research is unbalanced with respect to the deep learning model. In order to avoid overshooting of CNN training and to obtain a superior deep learning model, a data augmentation Tian, et al. [34] proposed was used to increase the number of samples.

Ultimately, the 28 raw data from the class I were augmented to 112. Every 7 original spectral data were divided into groups (grouped adjacent to DON index), and the data in each group were enhanced in pairs. The 109 raw data in the class II were expanded to 217. Every 3 original spectral data were divided into groups, and the data in each group were enhanced in pairs. The training and test sets were divided according to 7:3, with 548 samples in the training set and 234 samples in the test set after data enhancement. The distribution of samples in the test set was the same as that in the training set.

### 2.2. Data Pre-Processing Method

Conventional max-min normalization (MMN) is a method to normalize data by showing all factors in the whole data set x as values between 0 and 1 [35]. It determines the range of data by setting the minus between the maximum and minimum amounts acting as the numerator. With regard to the numerator, each element of x can be represented as a value between 0 and 1 by deducting the minimum value of the x element from each factor of x. Wavelet transform (WT), a new mathematical technique, has been shown to have fast computational and fast fading properties [36]. WT theory was extensively developed in the 1980s. It can be seen as a synthesis of ideas derived from physics (coherent states and reformation groups), pure mathematics (the study of Calderon-Zygmund operators) and engineering (sub-band coding). WT has emerged as a widespread tool for the analysis of signals. In addition to the two methods mentioned above, other pre-processing methods were also used in this study. First difference (FD) is a transformation of a time series consisting of a difference between adjacent periods, i.e., the latter period is subtracted from the former. The moving-average filtering (MAF) is a statistical rule based on which successive sampled data are considered as a queue of fixed length N. Following a new measure, the first of the above queue is deleted, the rest of the N-1 data are moved forward in sequence and the new sampled data are plugged in as the end of the new queue; the arithmetic operation is then carried out on this queue and the outcome is taken as the result of the current measurement.

### 2.3. Traditional Machine Learning Methods

SVM is a computational algorithm that assigns labels to objects through instance learning [37]. This algorithm has been widely applied for speech recognition, image recognition, text classification, face detection and error card detection [38]. Perceptron [39] is a binary linear model with a feature vector as input and a category as output; Perceptron acts as a hyperplane that divides data into positive and negative categories and is the most basic classifier in machine learning. Stochastic gradient descent (SGD), which is also referred to as stochastic approximation, describes the structure of certain simple iterations for solving random optimization and root searching problems [40]. The identifying features of SGD are very similar to those of gradient descent used for deterministic optimization. Each successive iteration in a recursion is identified by adding appropriately scaled gradient estimates to the previous iterations. Random forest (RF) is a supervised algorithm that uses an integrated learning method consisting of numerous decision trees, with the output being a consensus on the best answer to the problem [41]. K nearest neighbors (KNN) is a common algorithm used for supervised learning, which works by finding the K nearest training samples in the training set based on some distance given a test sample, and then predicting based on information from these K neighbors [42]. Decision tree [43] (DT) is a fundamental approach of classification and registration. A DT model is a dendritic structure that indicates the process of categorizing instances on the basis of features in a categorization issue. Naive bayes (NB) is a method of classification founded on Bayes’ theorem and the conditional independence assumption of features [44]. The joint probability distribution from input to output is first learnt from a known set of training items, assuming that independence between feature terms as a prerequisite. Input X is used to find the output Y that maximizes the posterior probability based on the learned model.

### 2.4. Convolutional Neural Network (CNN)

**Figure 2:**
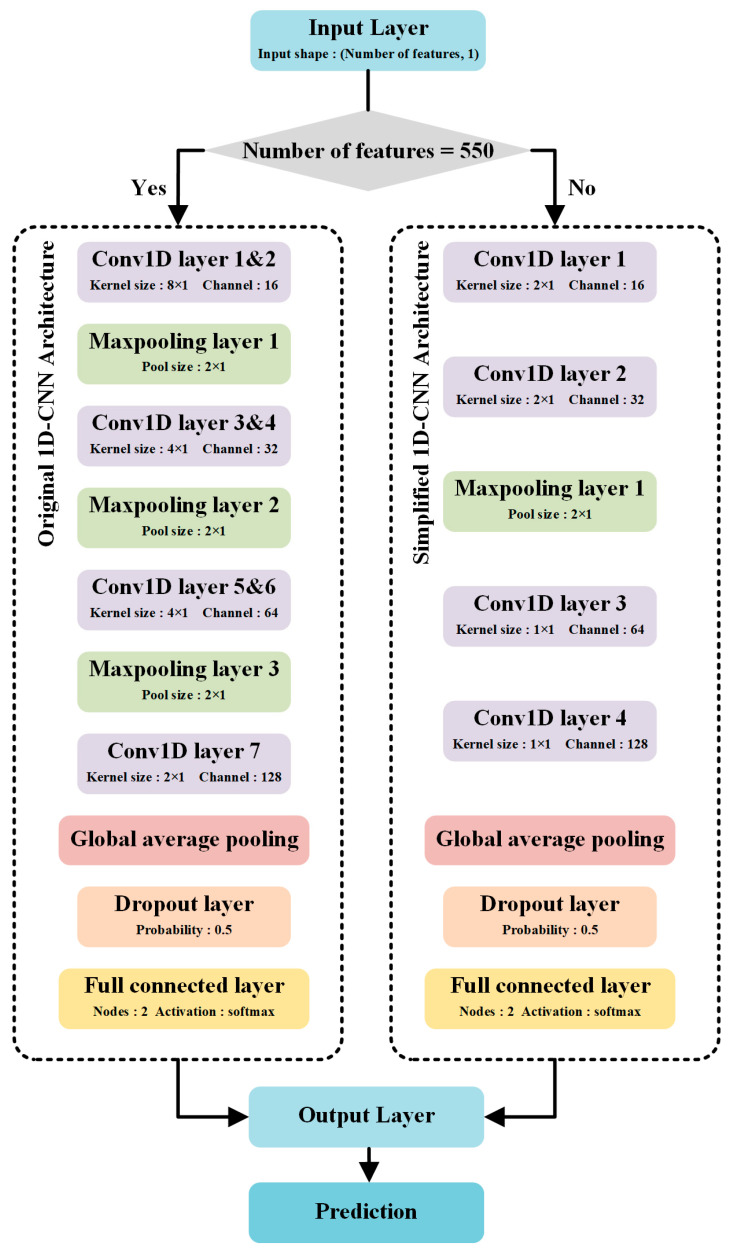
Diagram of the hyperspectral imaging (HSI) system.

The fundamental structure of a CNN is composed of a convolutional layer, an activation function layer, a pooling layer and a fully connected layer. The convolutional layer is primarily employed to select hidden features from the data. The feature map of the inputs data is available by means of a continuous sliding filter. In this study, two CNN architectures were proposed. The pool size was set to 2 × 1. As shown in Figure 2, the data is input into the original 1D-CNN when the number of features is 550. The original 1D-CNN includes one input layer, seven convolutional layers, three max pooling layers, one global average pooling layer, one dropout layer and one full connected layer. While the simplified 1D-CNN consists of one input layer, four convolutional layers, one max pooling layers, one global average pooling layer, one dropout layer and one full connected layer. The input layer is used for inputting spectral data and the output layer is used for prediction. In addition, a 2 × 1 max-pooling layer reduces the feature dimension by half, and the three maxpooling layers are used in the original model structure as shown in Figure 2. Such a structure can work normally when the input feature quantity is 550. When the number of features is less than a certain value, the maxpooling layer does not work and increases the calculation time. Therefore, a simplified model is proposed for training after feature band selection. In the simplified model, due to the reduction in the number of features, we have reduced the kernel size of the convolution layers and the number of maxpooling layers to adapt to the smaller number of features. Compared to the original model structure, a smaller kernel size means a smaller receptive field, so the original model structure has better feature extraction ability under 550 features. Therefore, it is chosen to retain both the original model and the simplified model for use in different feature quantity situations. It should be noted that the experiments before feature band selection in this paper are trained by the original model, and the subsequent experiments are completed by the simplified model.

### 2.5. Variable Selection Algorithm

CARS is an efficient tactic for the selection of the best combined of key wavelengths in multicomponent spectral data according to the “survival of the fittest” principle [45]. A subset of N variables is selected by N iterative ways through N number of sampled runs, with the final subset with the smallest root mean square error of cross-validation (RMSECV) value being selected as the best subset. CARS performs four consecutive steps in each sampling run, including Monte Carlo model sampling, EDF forced wave reduction, ARS competitive wave reduction and RMSECV computation of each subset. CARS achieves the selection of the optimal subset of wavelengths to some extent.

SPA [46] is a forward selection method that uses simple operations to minimize variable covariance in vector space, as a new variable selection strategy for multivariate calibration. SPA carries out a simple projected operation in vector space to get a useful subset of variables with minor covariances. Here is a summary of the main points. First, before selecting the starting vector in the n-dimensional space, set the maximum number of variables N to be selected (where n is an original number of variables). Afterwards, the vector of the higher projection is selected in the orthogonal subspace to become the starting new vector. At each iteration an orthogonal subspace is chosen so that only non-covariates are selected. The best initial variables and number of variables can be identified from the minimum root mean square error of validation (RMSEV) [47].

### 2.6. Model Evaluation

Performance of the 1D-CNN was assessed using a few parameters. False positives (FP), false negatives (FN), true positives (TP) and true negatives (TN) were computed and applied to generate metrics including confusion matrix, classification error, recall and precision and F1-score. The confusion matrix gives a direct indication of the model’s exact prediction results for each class. Precision is applied to assess the model’s overall performance. F1-score, classification error, precision and recall were employed to assess the model’s classification performance for each class. The calculation equations of each index are as follows:

(1) $$\mathrm{Precision}=\frac{\mathrm{TP}}{\mathrm{TP}+\mathrm{FP}}$$

(2) $$\mathrm{Recall}=\frac{\mathrm{TP}}{\mathrm{TP}+\mathrm{FN}}$$

(3) $$F1-\mathrm{score}=\frac{2\times \mathrm{Precision}\times \mathrm{Recall}}{\mathrm{Precision}+\mathrm{Recall}}$$

where TP is the number of samples from the target class that the model correctly found, FN is the number of samples from the target class that the model incorrectly found, and FP is the number of samples from other classes that the model incorrectly judged to be the target class TN is the number of samples from other classes that the model correctly found.

Python was utilized to preprocess the spectral data. Traditional machine learning models including SVM, SGD, RF and KNN were also implemented with Python. The proposed CNN architecture was constructed using program language Python 3.7. All software tools were carried out on a Win11 64-bit computer with Intel(R) Core™ i7-10750H CPU, 2.60 GHz, 2.59 GHz and 16 GB RAM (ASUS, China).

## 3. Results

### 3.1. Full Wavelength Models

The performance of the 1D-CNN model proposed was much better compared with other traditional machine learning algorithms, as illustrated in Table 1. The 1D-CNN achieved the highest precision of 89.41%, the recall of 0.8922 and the F1-score of 0.8911 when classifying the class (I, II) and the class III. It also performed best when classifying class I and class II with a Precision of 90.08%, a Recall of 0.8947 and a F1-Score of 0.8961. All models achieved a precision greater than 80% for one-step classification. In particularly, perceptron and SGD, only achieved 15.52% and 36.73% accuracy respectively. This could be attributed to the fact that these two models are not very powerful in classifying low levels of DON. In summary, it suggests that 1D-CNN is expected to replace ordinary machine learning algorithms when classifying DON levels.

### 3.2. Data Pre-Processing

Spectral preprocessing improved the classification performance of the 1D-CNN model. Different one-step spectral pre-processing methods such as MMN, MAF, WT were first used. As shown in Table 2, the WT achieved the highest precision (91.48%), recall (0.9138) and F1-score (0.9132) when classifying the class (I, II) and the class III. And it is worth noting that the filter used in this study is the Daubechies wavelet transform filter. While the FD performed best in classifying the first and second classes with a precision of 93.86%, a recall of 0.9368 and a F1-score of 0.9373.

**Figure 3:**
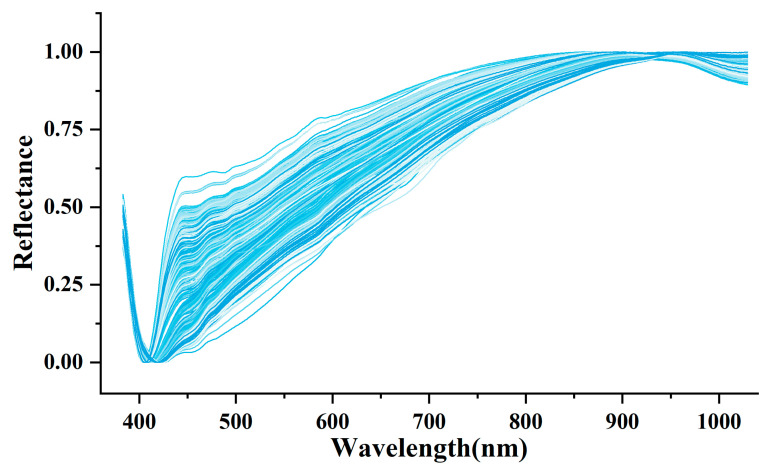
Reflectance of Max-Min Normalization and Moving-Average Filtering after the two-step pre-processing.

Figure 3 showed the reflectance spectra of MMN and MAF after the two-step pre-processing. The reflectance spectra were more concentrated around 420 nm and more dispersed around 450 nm after the two-step pre-processing. The results of the 1D-CNN model developed using two-step spectral pre-processing is shown in Table 3. The model on the basis of MAF-WT achieved the highest precision of 91.96%, the recall of 0.9181 and the F1-score of 0.9174 when classifying Class (I, II) and Class III, while the model based on WT-MMF performed best when classifying Class I and Class II with a precision of 95.05%, a recall of 0.9474 and an F1-score of 0.9479.

### 3.3. Feature Wavelength Selection

**Figure 4:**
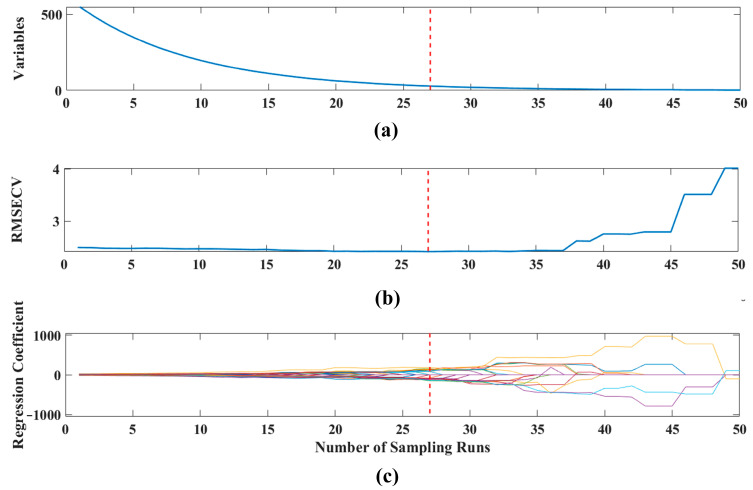
Selection of feature variable based on CARS method, (**a**) number of variables taken for sampling, (**b**) RMSECV values, and (**c**) the path of regression coefficients for each variable as the number of samples increases. The line (labelled with a dashed line) indicates the optimal point with the lowest RMSECV value.

Various variable selection algorithms were employed to recognize the characteristic wavelengths. The CARS-based wavelength selection process is depicted in Figure 4. There were 50 Monte Carlo sampling runs performed in CARS. Each variable’s contribution was assessed by cross-validation. As shown in Figure 4a, the number of sampling variables decreases as the number of sampling runs increases. The decrease is greatest at the beginning and then gradually decreases as the number of sampling passes increases. Through this approach, most of the redundant variables in the full spectral range (550 variables) are gradually eliminated. Figure 4b depicts the RMSECV values versus the number of sample runs. The best subset of characteristic variables was determined by the lowest RMSECV values produced in multiple sampling runs. It is evident that the RMSECV value decreases continuously until the number of sampling runs reaches 27, then the RMSECV value gradually increases. The minimum RMSECV value for the 22nd sampling run is marked with a red dashed line, indicating the combination of the characteristic variables. Figure 4c shows the regression coefficient paths for the 550 variables in different sampled runs. The values of the coefficients for some samples decrease to zero, while the values of the coefficients for some other variables increase. Based on the CARS algorithm, 28 of the 550 variables were selected (390.905, 392.015, 393.126, 403.128, 407.581, 413.152, 415.382, 431.022, 433.261, 456.825, 460.2, 569.42, 571.72, 584.39 606.346, 607.504, 608.662, 724.598, 728.15, 810.477, 815.287, 823.714, 924.577, 970.104, 971.339, 1014.733, 1018.467 and 1023.449 nm). Although the 28 selected wavelengths represent only 5.09% of all variables, they may still be too many to be any real use. Versatile spectral imaging systems for use in real-time monitoring require the smallest possible number of characteristic wavelengths with consistent detection precision.

**Figure 5:**
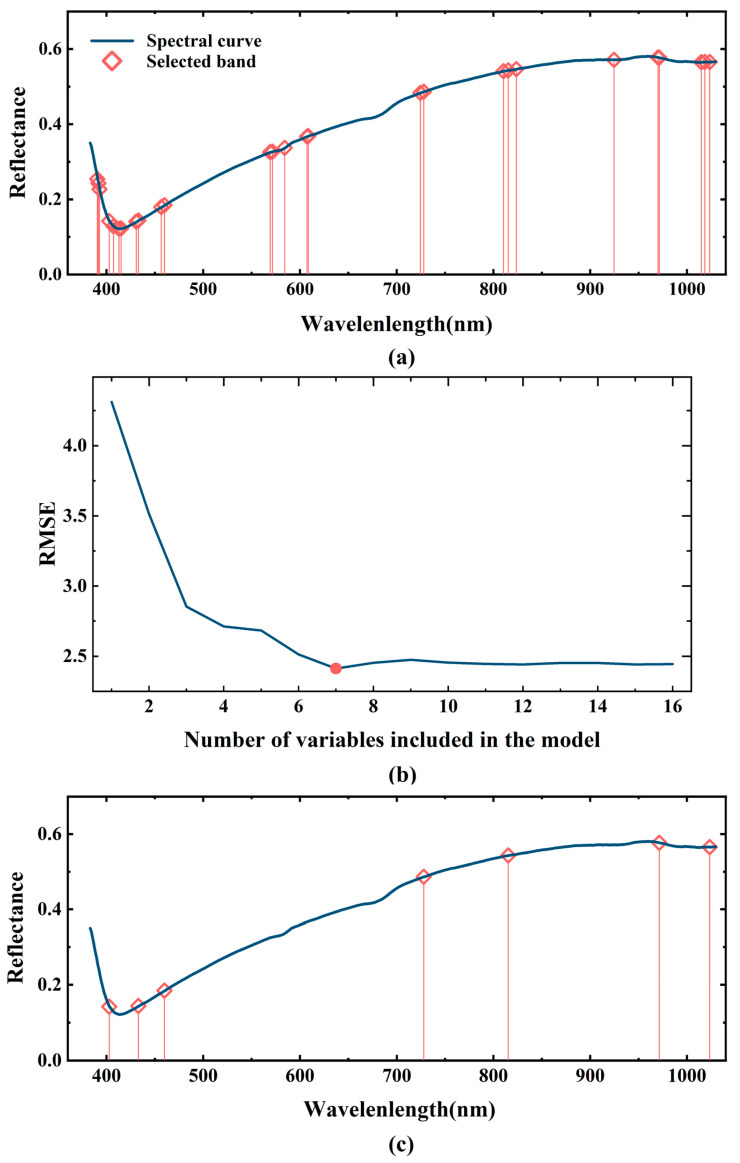
Characteristic wavelengths selected via CARS (**a**) and CARS-SPA (**c**), and the RMSE values with the increase of the number of wavelengths in the model (**b**).

An evaluation of the 28 wavelengths selected by CARS was re-evaluated using the SPA algorithm in order to optimize the selected variables. It can be clearly observed from Figure 5 that the RMSE values show a decreasing trend with the increase of the number of wavelengths. When the number of wavelengths is greater than 7, the change in RMSE values is no longer significant.

### 3.4. Model Optimization

The simplified 1D-CNN models were constructed with the selected feature bands. The classification results of the models using CARS, SPA and CARS-SPA are shown in Table 4, based on the results of the pre-processing of the raw spectral data by MMN, WT, MAF, etc. When only one feature variable selection method is considered to extract feature bands, the number of feature bands obtained is more than the variables selected when the two methods of CARS and SPA are combined. The built model worked best with the two-step preprocessing method (combination of WT and MMN) combined with the two-step feature band selection method (combination of CARS and SPA). The precision of the model built using seven feature bands was slightly lower than that of the model built using 28 bands, but the results of both models were almost equivalent. Based on WT-MMN-CARS-SPA, seven feature bands were finally identified. The optimized 1D-CNN model achieved a precision of 88.38%, a recall of 0.8836 and an F1-score of 0.8829 when classifying class (I, II) and class III. Furthermore, a precision of 89.81%, a recall of 0.8981 and an F1-score of 0.8955 were achieved when classifying class I and class II. To sum up, WT-MMN-CARS-SPA not only lowers the original data complexity, but also keeps the precision of the model. The results indicate that CARS-SPA is preferable for the selection of characteristic wavelengths.

### 3.5. Comparison of Optimized Models

After using WT-MMN to perform two-step preprocessing on the original data and using CARS-SPA to obtain seven feature bands, the effects of different machine learning methods established by using the selected feature bands to classify the data were compared, as shown in Table 5. Compared with the proposed CNN method, the classification results of the other machine learning methods were much worse. LR had the worst performance with only 29.21% precision in the first step of classification and 39.22% precision in the second step of classification. This suggests that LR is not suitable for classifying and predicting 1D data. Both the SVM and perceptron models had a precision above 79% for classification of class (I, II) and class III, but below 40% for further classification. RF achieved a precision of 84.29% for one-step classification and 86.92% for two-step classification, but neither was as good as the simplified 1D-CNN model.

**Figure 6:**
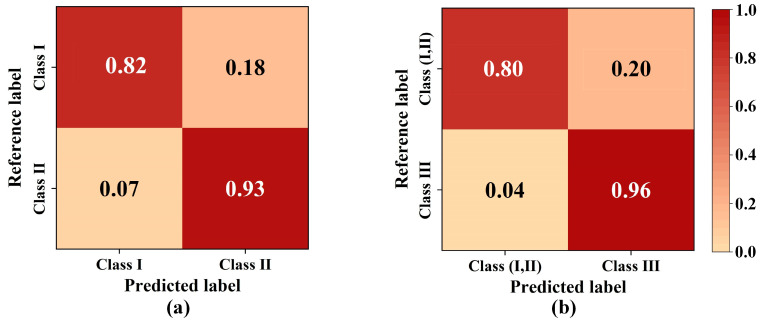
Confusion matrices for first (**a**) and second (**b**) step classification based on the optimized 1D-CNN model.

The final optimized 1D-CNN model also achieved a high accuracy rate on the test dataset. The accuracy of the 1D-CNN model was 80% and 96% when initially classifying class (I, II) and class III for testing, as shown in Figure 6a. As shown in Figure 6b, the accuracies were 82% and 93% for classifying class I and class II separately. The accuracy at this point is less than that in Figure 6, due to the fact that some of the relevant information is lost through feature band extraction, which is in line with the general rule.

## 4. Discussion

In general, the optimized 1D-CNN model in this paper can classify and predict spectral data. Compared with the traditional models, the final prediction accuracy of the proposed method is 88.38% with a recall of 0.8836 and an F1-score of 0.8829 when classifying Class I, II and Class III. While a precision of 89.81%, a recall of 0.8981 and a F1-score of 0.8955 were achieved when classifying class I and class II. In order to obtain more accurate training results, data augmentation was performed on three levels of data. In the end, the class I was enhanced from 28 to 112, class II from 109 to 217. And the class III was reduced from 819 to 453, removing spectral data with DON levels greater than 14 mg/kg. A higher resolution and predictive power for lines with lower DON content would be helpful for selection purposes. In breeding programs, samples of greater than 14 mg/kg of actual barley grain may be discarded. These samples are not intended for human use for grain buyers, but can be blended with samples containing lower levels of DON to achieve tolerable levels for animals on farms, particularly for ruminants that can cope with higher levels of mycotoxins. In contrast to pigs, which are very DON-sensitive, tolerances in ruminants such as dairy cattle are comparatively high because micro-organisms in the rumen can convert these toxins into the lower virulence deep oxygen DON [48]. And the enhanced spectral data are kept at a certain scale rather than averaged to prevent over-fitting.

Prior studies have explored the possibility of coupling hyperspectral imaging with selected feature variables to evaluate DON in barley grains and to categorize grain samples into various categories depending on DON levels [8]. Based on the seven wavelengths selected by CARS and iterative selection of SPA (ISSPA), partial least squares discriminant analysis (PLSDA) distinguished barley grains with low DON levels (<1.25 mg/kg) to those containing high levels (comprising 1.25–3 mg/kg, 3–5 mg/kg and 5–10 mg/kg) with a Mathews correlation coefficient (M-R) of 0.931 in cross-validation. Compared to the above-mentioned studies, the proposed method chooses different classification intervals and achieves the same high level of accuracy. So far as we know, this is the first time that deep learning has been applied to the online detection of wheat plague level grading In this study, the proposed 1D-CNN can be used to efficiently analyze spectral data. The convolutional layers in 1D-CNN are specially designed to extract concealed features in the spectrum more accurately and efficiently than traditional machine learning methods.

The characteristic wavelength model can achieve prediction precision similar to that of the full band model. In the study, the 28 variables selected by CARS (390.905, 392.015, 393.126, 403.128, 407.581, 413.152, 415.382, 431.022, 433.261, 456.825, 460.2, 569.42, 571.72, 584.39 606.346, 607.504, 608.662, 724.598, 728.15, 810.477, 815.287, 823.714, 924.577, 970.104, 971.339, 1014.733, 1018.467, 1023.449 nm) lie in the visible and NIR spectral ranges within the visible and NIR spectra, suggesting that the cyan region of the spectra may not contain characteristic information relevant to the DON content. After removing 21 of the 28 wavelengths, the remaining seven feature wavelengths selected by CARS-SPA (403.128, 433.261, 460.2, 728.15, 815.287, 971.339, and 1023.449 nm) were used to develop a 1D-CNN model to predict DON content, yielding very similar accuracy as using 28 variables. This indicated the redundant information provided by the 21 variables that were removed. With the majority of the redundant information removed, the improved model still retained a high prediction precision and greatly facilitated the speedy determination of DON in barley kernel samples.

However, the classification performance was insufficient. The errors in prediction may be due to the low DON concentration of the barley samples used in this study. After feature band extraction using CARS-SPA, the accuracy was reduced due to the removal of some of the bands containing valid spectral information. At the same time, however, the model was simplified and the efficiency was improved. More efficient methods are needed in the future to analyze hyperspectral data with high complexity. The processing of the initial data can also be further improved, and the replacement of wavelet transform filters and changes to filter parameters will be further attempted in the future to optimize the whole process in order to develop more efficient and accurate models. In addition to this, laboratory environment can also affect the accuracy of the model. For the best possible precision of the model, changes in the external environment, like temperature and humidity, should be avoided in the online detection of DON levels that affect the spectral acquisition. Hence in the future, further experiments will be carried out on different varieties and growing environments and portable or mobile equipment will be developed to carry out experiments under different field conditions.

## 5. Conclusions

A combination of HSI technology and 1D-CNN was used in this study to classify DON levels in barley kernels. The data were subjected to one-step and two-step pre-processing after the enhancements as the physicochemical values of DON were too small. In order to eliminate the collinearity variable in the original spectrum and simplify the model, the feature wavelength extraction was performed. CARS was used to search hundreds of variables for characteristic wavelengths that may be related to the DON levels in the particulate samples, and these identified wavelengths are then re-optimized by the SPA. The results showed that the optimized CARS-SPA-CNN model was successfully used to distinguish barley samples with low levels of DON fortification (≤5 mg/kg) from those with high levels of DON fortification (>5 mg/kg) with a precision of 89.41%. Further, when classifying class I (DON: >0.19 mg/kg and ≤1.25 mg/kg) and class II (DON: >1.25 mg/kg and ≤5 mg/kg), the accuracy was 89.81%. Compared with other models, the proposed method showed the highest precision. However, the regression performance still has the potential to be improved, the processing of the initial data can be further improved and the laboratory environment can also affect the accuracy of the model. Based on this approach, future studies will validate the reliability of the technique using samples with a wide range of DON levels from different regions, seasons and experiments. It is certain that the optimized CARS-SPA-CNN model in this study will have a broad application in the spectral analysis of agricultural crops.

## Figures and Tables

**Table 1:** Precision, recall, and F1-score of the classification for different models.

Models	Class (I, II) and Class III			Class I and Class Ⅱ		
	Precision (%)	Recall	F1-Score	Precision (%)	Recall	F1-Score
1D-CNN	89.41	0.8922	0.8911	90.08	0.8947	0.8961
SVM	86.83	0.8681	0.8674	81.68	0.7374	0.6951
LR	84.33	0.8340	0.8299	78.92	0.6768	0.5984
Perceptron	83.57	0.8340	0.8322	15.52	0.3939	0.2226
SGD	81.17	0.7489	0.7454	36.73	0.6061	0.4574
RF	81.68	0.8170	0.8169	81.82	0.8182	0.8149
DT	77.81	0.7787	0.7769	68.15	0.6869	0.6824
NB	59.89	0.5277	0.4998	49.40	0.4444	0.4388

SVM, Support Vector Machine; LR, Logistic Regression; SGD, Stochastic Gradient Descent; RF, Random Forest; Decision Tree, DT; Naive Bayes.

**Table 2:** Precision, Recall, and F1-score for prediction of spectral data with 1D-CNN based on different one-step pre-processing methods.

One-Step Pre-Processing Method *	Class (I, II) and Class III			Class I and Class Ⅱ		
	Precision (%)	Recall	F1-Score	Precision (%)	Recall	F1-Score
None	89.41	0.8922	0.8911	90.08	0.8947	0.8961
FD	90.20	0.9009	0.9	93.86	0.9368	0.9373
MMN	89.72	0.8966	0.8958	93.39	0.9263	0.9275
MC	89.41	0.8922	0.8911	88.84	0.8842	0.8854
MAF	89.64	0.8966	0.8962	90.62	0.9053	0.9056
MSC	90.60	0.9052	0.9045	89.86	0.8842	0.8865
SNV	90.13	0.9009	0.9002	86.15	0.8632	0.8614
Standardlize	89.67	0.8966	0.896	79.66	0.8	0.7974
VN	89.72	0.8966	0.8958	91.97	0.9158	0.9164
WT	91.48	0.9138	0.9132	90.62	0.9053	0.9056

* FD, First Difference; MMN, Max-Min Normalization; MC, Mean Centralization; MAF, Moving-Average Filtering; MSC, Multiplicative Scatter Correction; SNV, Standard Normal Variate; VN, Vector Normalization; WT, Wavelet Transform.

**Table 3:** Precision, Recall, and F1-score for prediction of spectral data with 1D-CNN based on different two-step pre-processing methods.

Two-Step Pre-Processing Method	Class (I, II) and Class III			Class I and Class Ⅱ		
	Precision (%)	Recall	F1-Score	Precision (%)	Recall	F1-Score
MMN-MAF	90.07	0.9009	0.9006	91.79	0.9158	0.9164
MMN-WT	89.20	0.8922	0.892	92.63	0.9158	0.9173
MAF-MMN	89.67	0.8966	0.896	90.91	0.9053	0.9062
MAF-WT	91.96	0.9181	0.9174	90.47	0.9053	0.9049
WT-MMN	90.51	0.9052	0.9049	95.05	0.9474	0.9479
WT-MAF	91.48	0.9138	0.9132	92.14	0.9158	0.9169

MMN, Max-Min Normalization; MAF, Moving-Average Filtering; WT, Wavelet Transform.

**Table 4:** Precision, Recall, and F1-score of the classification for different methods of extraction of feature bands.

Extraction of Feature Bands	Class (I, II) and Class III			Class I and Class Ⅱ		
	Precision (%)	Recall	F1-Score	Precision (%)	Recall	F1-Score
MAF-WT-CARS(39)	90.43	0.9009	0.8995	93.95	0.9263	0.9278
MAF-WT-SPA(31)	90.38	0.9004	0.899	72.53	0.7340	0.7275
WT-MAF-CARS(20)	89.97	0.8966	0.8971	72.13	0.7053	0.7105
WT-MMN-CARS(28)	90.95	0.9052	0.9038	92.98	0.9263	0.9271
WT-MMN-SPA(30)	91.57	0.9138	0.9130	91.79	0.9158	0.9164
WT-MMN-CARS-SPA(7)	88.38	0.8836	0.8829	89.81	0.8966	0.8955

MMN, Max-Min Normalization; MAF, Moving-Average Filtering; WT, Wavelet Transform; CARS, Competitive adaptive reweighted sampling; SPA, Successive projections algorithm.

**Table 5:** Precision, Recall, and F1-score of the classification based on different machine learning models.

Models	Class (I, II) and Class III			Class I and Class Ⅱ		
	Precision (%)	Recall	F1-Score	Precision (%)	Recall	F1-Score
1D-CNN	88.38	0.8836	0.8829	89.81	0.8966	0.8955
SVM	85.32	0.8170	0.8090	39.22	0.6263	0.4823
LR	29.21	0.5404	0.3792	39.22	0.6263	0.4823
Perceptron	79.52	0.6979	0.6592	39.22	0.6263	0.4823
SGD	83.18	0.7702	0.7533	72.22	0.6667	0.5798
RF	84.29	0.8340	0.8314	86.92	0.8687	0.8665
DT	79.53	0.7914	0.7891	72.88	0.7273	0.7279
NB	65.47	0.6553	0.6549	56.33	0.5657	0.5644

SVM, Support Vector Machine; LR, Logistic Regression; SGD, Stochastic Gradient Descent; RF, Random Forest; Decision Tree, DT; Naive Bayes.

## Data Availability

Data available on request due to privacy.
